# Genetic conditions of short stature: A review of three classic examples

**DOI:** 10.3389/fendo.2022.1011960

**Published:** 2022-10-21

**Authors:** Merlin G. Butler, Bradley S. Miller, Alicia Romano, Judith Ross, M. Jennifer Abuzzahab, Philippe Backeljauw, Vaneeta Bamba, Amrit Bhangoo, Nelly Mauras, Mitchell Geffner

**Affiliations:** ^1^ Department of Psychiatry & Behavioral Sciences, University of Kansas Medical Center, Kansas City, KS, United States; ^2^ Department of Pediatrics, University of Kansas Medical Center, Kansas City, KS, United States; ^3^ Pediatric Endocrinology, University of Minnesota Masonic Children’s Hospital, Minneapolis, MN, United States; ^4^ Department of Pediatrics, New York Medical College, Valhalla, NY, United States; ^5^ Department of Pediatrics, Nemours Children’s Health, Wilmington, DE, United States; ^6^ Department of Pediatrics, Thomas Jefferson University, Philadelphia, PA, United States; ^7^ Diabetes and Endocrine Center, Children’s Minnesota, Saint Paul, MN, United States; ^8^ Cincinnati Children’s Hospital Medical Center, University of Cincinnati College of Medicine, Cincinnati, OH, United States; ^9^ Division of Endocrinology, Children’s Hospital of Philadelphia; Department of Pediatrics, Perelman School of Medicine, University of Pennsylvania, Philadelphia, PA, United States; ^10^ Pediatric Endocrinology, Children's Health of Orange County (CHOC) Children’s Hospital, Orange, CA, United States; ^11^ Division of Endocrinology, Nemours Children’s Health, Jacksonville, FL, United States; ^12^ The Saban Research Institute, Children’s Hospital Los Angeles, Los Angeles, CA, United States; ^13^ Keck School of Medicine, University of Southern California, Los Angeles, CA, United States

**Keywords:** Noonan syndrome, Turner syndrome, Prader-Willi syndrome, short stature, growth hormone, genetics

## Abstract

Noonan, Turner, and Prader-Willi syndromes are classical genetic disorders that are marked by short stature. Each disorder has been recognized for several decades and is backed by extensive published literature describing its features, genetic origins, and optimal treatment strategies. These disorders are accompanied by a multitude of comorbidities, including cardiovascular issues, endocrinopathies, and infertility. Diagnostic delays, syndrome-associated comorbidities, and inefficient communication among the members of a patient’s health care team can affect a patient’s well-being from birth through adulthood. Insufficient information is available to help patients and their multidisciplinary team of providers transition from pediatric to adult health care systems. The aim of this review is to summarize the clinical features and genetics associated with each syndrome, describe best practices for diagnosis and treatment, and emphasize the importance of multidisciplinary teams and appropriate care plans for the pediatric to adult health care transition.

## Introduction

Noonan, Turner, and Prader-Willi syndromes are classical genetic conditions that share poor linear growth as a common feature. While the three syndromes share some characteristic features, each represents a vastly different genetic defect (sex chromosome aneuploidy, autosomal dominant mutation, and errors in genomic imprinting). These syndromes are all accompanied by comorbidities that require proper monitoring and treatment throughout a patient’s lifetime. Each syndrome is well-recognized and is backed by extensive published literature describing their features, genetic origins, and optimal treatment strategies. However, treatment of individuals may be complicated by diagnostic delays and lack of communication among healthcare specialists. Furthermore, insufficient information is available on natural history to guide the patient with Noonan, Turner, or Prader-Willi syndrome from infancy into adulthood.

This review delineates the clinical features and genetics associated with each disorder, details diagnosis and treatment methods, and discusses the importance of multidisciplinary care and proper transition from pediatric to adult health care systems.

## Noonan syndrome

Noonan syndrome (NS) is a congenital genetic disorder that occurs in 1 in every 1000 to 2500 live births (1–3). It is caused by germline gain-of-function RAS/MAPK (mitogen-activated protein kinase) pathway variants and is therefore classified as a RASopathy (4). RAS/MAPK signaling is typically involved in the cell cycle, proliferation, differentiation, growth, and senescence, with alterations in this pathway having significant effects on developmental processes (5). NS-causing gene variants are typically inherited in an autosomal dominant fashion, although other patterns, including *de novo* and autosomal recessive patterns, have been observed (3, 6).

### Features

Individuals with NS display several characteristic traits, including distinctive craniofacial features, growth abnormalities, cardiovascular and skeletal anomalies, cryptorchidism in males, bleeding disorders, and other comorbidities (1).

The distinctive facial characteristics associated with NS are prominent in infancy, change as the child ages, and become more subtle in adulthood (7). The head is large with a tall forehead and the eyes are prominent and widely spaced (hypertelorism), sometimes with unilateral or bilateral ptosis (2). Epicanthal folds and downward-slanting palpebral fissures are also somewhat common (8). As an individual with NS ages, the eyes become less prominent, the hair may become curly or wooly, and the face lengthens into a triangular shape (7). Other notable facial features can be found in **Table 1**.

**Table 1:** Clinical features and comorbidities associated with Noonan, Turner, and Prader-Willi syndromes.

Fetal Characteristics		
Noonan	Turner	Prader-Willi
• Polyhydramnios •Pleural effusion •Increased nuchal translucency •Cystic hygroma •Hydrothorax	•Lymphedema (97%) •Polyhydramnios •Pleural effusion •Increased nuchal translucency •Cystic hygroma •Oligohydramnios •Left-sided obstructive cardiac anomalies	•Hypotonia (94%) •Reduced fetal movement (88%) •Polyhydramnios (34%)
**Facial Features**		
**Noonan**	**Turner**	**Prader-Willi**
**Head and Neck** •Low-set, posteriorly rotated ears (90%) •Low posterior hairline (68%) •Short neck (elongated in adulthood) with excess skin/webbing (55%) •Curly or wooly hair in adulthood (33%) •Wispy hair (10%) •Large head with a tall forehead and small face •Short, broad nose with a depressed root and full tip **Eyes** •Ophthalmologic problems (94%) Strabismus (48%-63%) Amblyopia (33%) Refractive errors Anterior segment changes •Down-slanted palpebral fissures with epicanthal folds (68%) •Ptosis (42%) •Hypertelorism •Telecanthus **Mouth** •Full upper lip with a deeply grooved philtrum (95%) •High-arched palate (>55%) •Micrognathia (33%-43%)	**Head and Neck** •Wide neck (40%) •Low posterior hairline (40%) •External ear abnormalities, including low-set ears and protruding pinnae (34%) •Webbed neck (25%) **Eyes** •Strabismus (25%-35%) •Ptosis (16%) •Epicanthal folds (11%) •Hypertelorism (3%) •Down-slanted palpebral fissures **Mouth** •Micrognathia (60%) •High-arched, narrow palate (35%)	**Head and Neck** •Narrow forehead (75%) •Elongated skull •Small, upturned nose **Eyes** •Almond-shaped, elongated eyes (75%) •Strabismus (60%-70%) •Up-slanting palpebral fissures **Mouth** •High-arched palate •Micrognathia •Downward-turned corners of mouth •Thin upper lip •Enamel hypoplasia
**Growth Characteristics**		
**Noonan**	**Turner**	**Prader-Willi**
•Short stature (~80%) •Feeding problems/poor suck (76%) •GH deficiency (37%-45%) •GH resistance	•Short stature (up to 95%) •GH resistance	•GH deficiency (40%-100%) •Short stature (90%) •Feeding problems/poor suck
Cardiovascular		
Noonan	Turner	Prader-Willi
•CHD (80%) •Bleeding disorders (30%-65%) •Intrinsic pathway abnormalities (50%) •Abnormal electrocardiographic pattern (50%) •PS (40%) •Factor deficiencies (FVIII, FXI, FXII), platelet defects (33%) •HCM (20%) •Atrioventricular canal defects (15%) •Atrial septal defect (6%-10%) •Left-sided obstructive lesions	•Elongated transverse aortic arch (50%) •Hypertension (50%) •Aortic dilation/aneurysm(3%-42%) •Prolonged QT interval (21%-36%) •Bicuspid aortic valve (15%-30%) •Aortic coarctation (7%-18%) •Aortic valve stenosis (4%-16%) •Mitral valve regurgitation or prolapse (1%-9%) •Stroke •Coronary artery disease •Hypoplastic left heart syndrome •Aortic insufficiency •Increased myocardial workload •Arrhythmias	•Right-sided heart failure
**Reproductive**		
**Noonan**	**Turner**	**Prader-Willi**
•Cryptorchidism (80% of males) •Pubertal delay •Gonadal dysfunction in males; normal fertility in females	•Primary ovarian insufficiency (95%-98%) •Pubertal delay (or absence) (85%) •Hypergonadotropic hypogonadism	•Central and/or primary hypogonadism (100%) •Cryptorchidism (80%-100% of males) •Pubertal delay (or absence)
**Renal and Endocrine**		
**Noonan**	**Turner**	**Prader-Willi**
•Horseshoe kidney •Duplicated collecting system •Renal pelvis dilation •Solitary kidney	•Autoimmune hypothyroidism (24%) •Duplicated collecting system (5%-15%) •Horseshoe kidney (10%) •Ectopic kidney (<1%) •Elevated gonadotropins •Kidney hypoplasia	•Central adrenal insufficiency (10%) •Central hypothyroidism (10%)
**Metabolic**		
**Noonan**	**Turner**	**Prader-Willi**
	•Glucose intolerance (15%-50%) •Obesity (15%) •Type II diabetes (10%) •Metabolic syndrome •Reduced insulin sensitivity •Gluten-sensitive enteropathy (celiac disease)	•Obesity (94%-100%) •Metabolic syndrome (38%) •Type II diabetes (25%) •Reduced glucose tolerance •Insulin resistance
Musculoskeletal		
Noonan	Turner	Prader-Willi
•Inferior pectus excavatum (95%) •Superior pectus carinatum (95%) •Joint hypermobility (50%) •*Cubitus valgus* (50%) •Scoliosis (10%-15%) •*Talipes equinovarus* (10%-15%) •Cervical spine fusion (2%) •Radio-ulnar synostosis (2%) •Osteopenia •Kyphosis •*Spina bifida* •Vertebral and rib abnormalities •*Genu valgum*	•Kyphosis (40%-75%) •Scoliosis (12%-59%) •*Cubitus valgus* (50%) •Short 4th metacarpal (35%) •*Genu valgum* (35%) •Pectus excavatum (20%) •Osteoporosis •Hip dysplasia •Radio-ulnar synostosis (Madelung deformity)	•Scoliosis (40%) •Hip dysplasia (10%-20%) •Osteopenia •Osteoporosis •Small hands and feet
Neurocognitive Characteristics		
Noonan	Turner	Prader-Willi
•ADHD (40%-50%)/ASD (30%) •Learning difficulties/intellectual disability (10%-40%) •Behavioral problems •Neurocognitive issues •Memory deficits	•Behavioral problems (25%) •ADHD (24%)/ASD •Learning difficulties (23%)/intellectual disability (10%) •Neurocognitive issues	•Behavioral problems (90%) •Compulsive tendencies (60%) •Learningdifficulties/intellectual disability (40%) •ASD (12%-41%) •Neurocognitive issues
**Other Clinical Features**		
**Noonan**	**Turner**	**Prader-Willi**
•Splenomegaly (52%) •Hearing loss (25%) •Disordered lymphatic development (20%) •Seizures (10%-13%) •Chylothorax (10%) •Peripheral lymphedema (5%-10%) •Widely-spaced nipples •Increased cancer rates •Lymphangiectasis •Abnormal lymphatic vessels	•Elevated liver enzymes (transaminitis) (50%-80%) •Sensorineural hearing loss (60% of adults) •Widely-spaced nipples (30%) •Nail hypoplasia (10%) •Celiac disease (4%-6%) •Inflammatory bowel disease (2%-3%) •Increased cancer rates •Lymphatic hypoplasia or aplasia •NAFLD/hepatosteatosis	•Seizures (10%-20%) •Sleep apnea
**References**		
**Noonan**	**Turner**	**Prader-Willi**
(1, 2, 8–18)	(19–28)	(29–42)

ADHD, attention deficit hyperactivity disorder; ASD, autism spectrum disorder; CHD, congenital heart disease; GH, growth hormone; HCM, hypertrophic cardiomyopathy; NAFLD, nonalcoholic fatty liver disease; PS, pulmonary stenosis.

**Figure 1:**
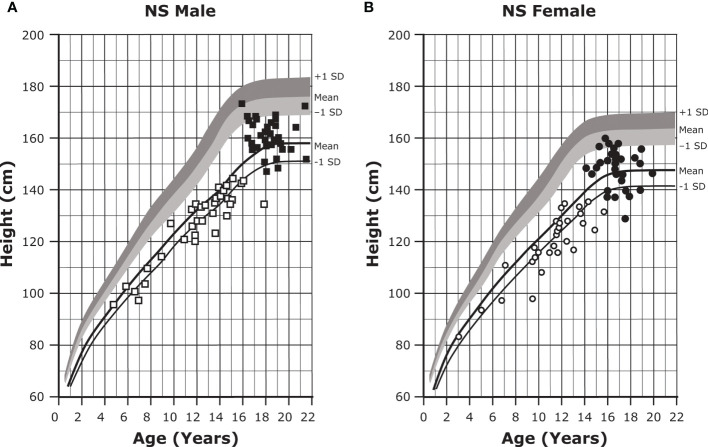
Growth curves for patients with NS. Heights (mean and -1 SD) of males **(A)** and females **(B)** with NS are shown with solid lines and overlaid on the normative percentile ranges, with normative mean to +1 SD in dark shading and normative mean to -1 SD in light shading. Also depicted are the heights of 35 males and 30 females with NS at the start of GH treatment (open symbols) and at final height (solid symbols). Adapted from Romano AA et al. J Clin Endocrinol Metab 2009;94(7):2338-2344. GH, growth hormone; NS, Noonan syndrome.

About 80% of individuals with NS will exhibit short stature (**Figures 1A, B**) (1, 43, 44). Although abnormal growth is a characteristic feature of NS, some individuals will display normal growth patterns and adult height. Birth weight and length tend to be within a normal range (45, 46); however, there is a subsequent deceleration of height and weight to -2.0 SD or less (43, 44). Mean adult heights range from -2.3 to -2.5 SD for males and -2.1 to -2.2 SD for females based on height references from four different patient populations (43, 44, 47, 48). Puberty is commonly but not universally delayed in both sexes and is associated with a reduced growth spurt (1, 8). There is a mean bone age delay of about two years, and reports of growth hormone (GH) secretory dynamics are inconsistent; various studies have reported GH deficiency (37% to 45% of individuals), neurosecretory dysfunction, and even normal GH secretion (1). Insulin-like growth factor I (IGF-I) concentrations are typically low and are significantly lower in individuals with *PTPN11* gene variants (1).

Most patients with NS display cardiac abnormalities (**Table 1**), including congenital heart disease (CHD) in about 80% of patients and hypertrophic cardiomyopathy (HCM) in about 20% of patients (16, 49). A broad spectrum of right- and left-sided cardiac lesions have been described in this condition. Pulmonary stenosis (PS), the most common manifestation of CHD, is present in about 40% of patients. Approximately 60% of PS cases are mild, with 10% presenting as moderate and 30% presenting as severe; 25% to 35% of patients with PS display a dysplastic valve (1, 16). Mild PS typically does not progress beyond early infancy, may resolve on its own within the first few months of life, and requires no intervention (50, 51). Beyond the age of 6 months, mild PS is often characterized as a static lesion (51). Fifty percent of patients with NS display an unusual electrocardiographic pattern, even in the absence of any other cardiac lesion (1).

Most individuals display superior pectus carinatum and inferior pectus excavatum, while 50% may display joint hypermobility or hypotonia (1, 8). Less common skeletal issues can be found in **Table 1**.

Unilateral and bilateral cryptorchidism have been reported in about 80% of male patients (1, 2, 8).

Disordered bleeding has been reported in patients with NS and can range from mild (easy bruising, menorrhagia) to severe (significant bleeding during surgical procedures) (1, 8). Several coagulation factor deficiencies (typically factors VIII, XI, or XII), platelet dysfunction, and thrombocytopenia have been described in individuals with NS (1, 16, 52–54). About 50% of individuals with NS have abnormalities in the intrinsic coagulation pathway and 65% display abnormal bruising or bleeding tendencies (2). Variants in the *PTPN11* gene, which encodes the SHP2 protein, may be particularly associated with bleeding problems. It should be noted that these defects are not necessarily correlated with the bleeding phenotype of patients with NS, and the explanation for bleeding risk remains incompletely understood. Recently, a study of patients with NS and NS with multiple lentigines and two *PTPN11* variant-driven NS mouse models revealed that platelet signaling defects are involved with NS-associated thrombopathy (55), providing insight into the bleeding effects of gain and loss-of-function *SHP2* variants.

### Other comorbidities

In addition to the five main features of NS—the characteristic facies, growth abnormalities, cardiovascular and skeletal anomalies, cryptorchidism in males, and bleeding disorders—there are many other comorbidities. These comorbidities are variable and may depend on gene variant associations (**Table 2**). No phenotype is exclusive to a single variant; however, some phenotypes may be more common in association with a particular genotype, as discussed below.

**Table 2:** Genotypic-phenotypic correlations in NS.

Genes involved	Frequency of cases	Most common features	References
*PTPN11*	50%	•Short stature •Chest deformity •Characteristic facial features •PS (45%-70% of patients) •Atrial septal defects •Less likely to have HCM •Hematologic abnormalities •Low IGF-I •No or mild cognitive impairment (especially in association with *N308S* variant) •Development of malignancies •Potentially elevated risk of hearing loss •More common in familial versus sporadic NS cases •Cryptorchidism	(1, 2, 9, 11, 16, 56–63)
*SOS1*	20%	•CFC syndrome–like skin findings (keratosis pilaris, sparse hair, curly hair, sparse eyebrows) •Less likely to have short stature •No or mild cognitive impairment •Ectodermal abnormalities •PS (~83% of patients) •Giant cell lesions	(1, 2, 11, 16, 58, 59, 63–65)
*LZTR1*	8%	•Predisposition to schwannomas	(11, 66)
*KRAS*	5%	•Severe learning issues and developmental delays •HCM •Possibly craniosynostosis	(11, 67–69)
*RIT1*	4%-9%	•Perinatal abnormalities (e.g., polyhydramnios) •High birth weight •Relative macrocephaly •CHD (94%) HCM (71%) PS (65%) •Increased ectodermal findings (including curly hair, hyperpigmentation, wrinkled palms and soles) •Less likely to have intellectual disability •Less likely to have short stature and chest deformities	(11, 16, 63, 70–75)
*SOS2*	4%	•Ectodermal defects •Infrequent short stature •Lymphatic abnormalities (>50%)	(11, 63)
*RAF1*	3%-17%	•HCM (95%)	(11, 16, 76)
*BRAF*	2%	•Short stature •Dysmorphic facial features •Skeletal anomalies •Hypotonia •Mild to moderate cognitive defects	(11, 77)
*MAP2K1*	<2%	•Increased ectodermal findings •Overall milder phenotype	(11, 72, 78)
*NRAS*	<1%	•Variable	(11, 79, 80)
*MRAS*	<1%	•Increased prevalence of HCM	(63, 81)
*A2ML1*	<1%	•Developmental delays	(11, 82)
*SHOC2*	NR	•Loose anagen hair •Mitral valve and cardiac septal defects •GH deficiency •Ectodermal abnormalities with darkly pigmented ichthyotic skin •Hypernasal voice •Developmental issues with hyperactivity	(1, 11, 83)

A2ML1, α2-macroglobulin-like-1; BRAF, V-Raf murine sarcoma viral oncogene homolog B1; CFC, cardiofaciocutaneous; CHD, congenital heart disease; GH, growth hormone; HCM, hypertrophic cardiomyopathy; IGF-I, insulin-like growth factor I; KRAS, kirsten rat sarcoma viral oncogene homolog; LZTR1, leucine-zipper-like transcription regulator 1; MAP2K1, mitogen-activated protein kinase kinase 1; MRAS, muscle RAS oncogene homolog; NR, not reported; NRAS, neuroblastoma rat sarcoma viral oncogene homolog; NS, Noonan syndrome; PS, pulmonary stenosis; PTPN11, protein-tyrosine phosphatase, non-receptor type 11; RAF1, v-raf-1 murine leukemia viral oncogene homolog 1; RIT1, Ric-like protein without Caax motif 1; SHOC2, SHOC2 leucine-rich repeat scaffold protein; SOS1, Son of Sevenless homolog 1; SOS2, Son of Sevenless homolog 2.

#### Gastrointestinal

Feeding problems have been described in up to 76% of infants with NS and appear to be associated with delayed developmental milestones and worse long-term outcomes (8, 84). About 24% may require tube feeding in early life. Most often, feeding problems are attributable to poor suck, gastroesophageal reflux, and/or delayed gastric emptying and typically resolve by 1 to 2 years of age (8).

#### Genitourinary

Renal issues (**Table 1**) occur in a minority (approximately 10%) of patients with NS and are typically of little clinical significance. Gonadal dysfunction in males with NS may be due to altered spermatogenesis (specifically Sertoli cell dysfunction) rather than cryptorchidism (1, 11, 85). Males with NS have reduced fertility; however, the exact frequency with which this occurs remains unknown (3, 86). Females exhibit normal fertility rates (1).

#### Lymphatic

Approximately 20% of patients with NS have manifestations of disordered lymphatic development. This most frequently presents as peripheral lymphedema in infancy and tends to resolve within the first few years of life, although it can recur in adolescence or adulthood. Lymphatic abnormalities can also present as lymphangiectasis or development of abnormal lymphatic vessels, particularly in the thoracic region (1). Some individuals with NS may display postoperative or spontaneous chylothorax (approximately 10% of cases) (16, 87, 88).

#### Hematologic/oncologic

Besides disordered bleeding, hematologic comorbidities include splenomegaly, which may cause thrombocytopenia and is seen by ultrasound in up to 52% of individuals with NS (89). Splenomegaly may be isolated, associated with hepatomegaly, and/or caused by NS/myeloproliferative disorder (NS/MPD). NS/MPD, typically seen in infants, is characterized by leukocytosis with monocytosis, thrombocytopenia, and hepatosplenomegaly. The clinical appearance of NS/MPD is similar to that of juvenile myelomonocytic leukemia, but infants with NS/MPD typically have a favorable prognosis and will remain stable or improve without specific therapy. Certain studies have suggested that patients with NS display elevated rates of malignancy due to hyperactivated RAS/MAPK signaling. Several case studies have reported specific malignancies, including gliomas, dysembryoplastic neuroepithelial tumors, and hematologic malignancies in patients with NS (2, 90–92). A Dutch study demonstrated a link between NS-associated *PTPN11* variants and increased cancer rates (93), while a recent German study reported significantly increased childhood cancer rates in patients with NS regardless of the underlying variant (94).

#### Neurologic/cognitive/psychological

NS-associated neurologic, cognitive, and psychological/behavioral issues are highly variable in frequency and severity and can significantly affect quality of life (1). Motor milestones are often delayed, with one study reporting that, on average, patients with NS sat unassisted at 10 months, walked at 21 months, and spoke in two-word sentences at 31 months (1, 8). Children with NS can have problems with verbal articulation and may benefit from early intervention with speech therapy (95, 96).

Individuals with NS typically display normal levels of intelligence, although 10% to 40% of patients may require some level of special education (1, 15, 18). The heterogeneity in cognitive abilities among individuals with NS may be ascribed to differences in the underlying molecular variant responsible for the condition (59) (**Table 2**).

Structural brain abnormalities are infrequent but may include hydrocephalus and Arnold-Chiari malformation type I (1). Patients with NS also appear to have smaller caudate, putamen, and pallidum volumes compared with controls (15). Recurrent seizures have been reported in 13% of patients (8).

Sensorineural issues may also be present, including ocular problems (94% of patients), hearing loss, and peripheral neuropathy (1, 2, 8).

Psychological and psychosocial issues associated with NS may include increased clumsiness, stubbornness, and irritability and weaker social skills compared with unaffected siblings (1, 97). Children with RASopathies, particularly NS, often display symptoms associated with attention deficit hyperactivity disorder (ADHD) and autism spectrum disorder (ASD), including inattention, poor impulse control, and hyperactivity, although they may not always meet the stringent criteria necessary for an official diagnosis (12, 13, 15, 17, 97). Language impairments are more frequent in children with NS and are associated with a higher risk of reading and spelling difficulties (98).

Those with NS may also experience poor body image, self-esteem issues, anxiety, and depression (1, 17). It has been suggested that some of these social issues may be linked to poor verbal development (95). Even into adulthood, individuals with NS may have difficulty maintaining satisfying social relationships and may report low-to-moderate levels of overall life satisfaction (17).

### Diagnosis

Early diagnosis is crucial for individuals with NS because each patient will require a unique treatment plan and will have a different prognosis and risk of recurrence (1). These authors suggest that screening for NS be added to newborn screening (NBS) programs to ensure rapid and early diagnosis.

Prenatally, NS may be suspected based on polyhydramnios, abnormalities of the lymphatic system (e.g., increased nuchal translucency, cystic hygroma, pleural or pericardial effusion, ascites, hydrops fetalis), and cardiac and/or renal abnormalities. An abnormal maternal blood triple screen test (for alpha-fetoprotein, human chorionic gonadotropin, and unconjugated estriol) may also be an indicator of NS (3).

**Figure 2:**
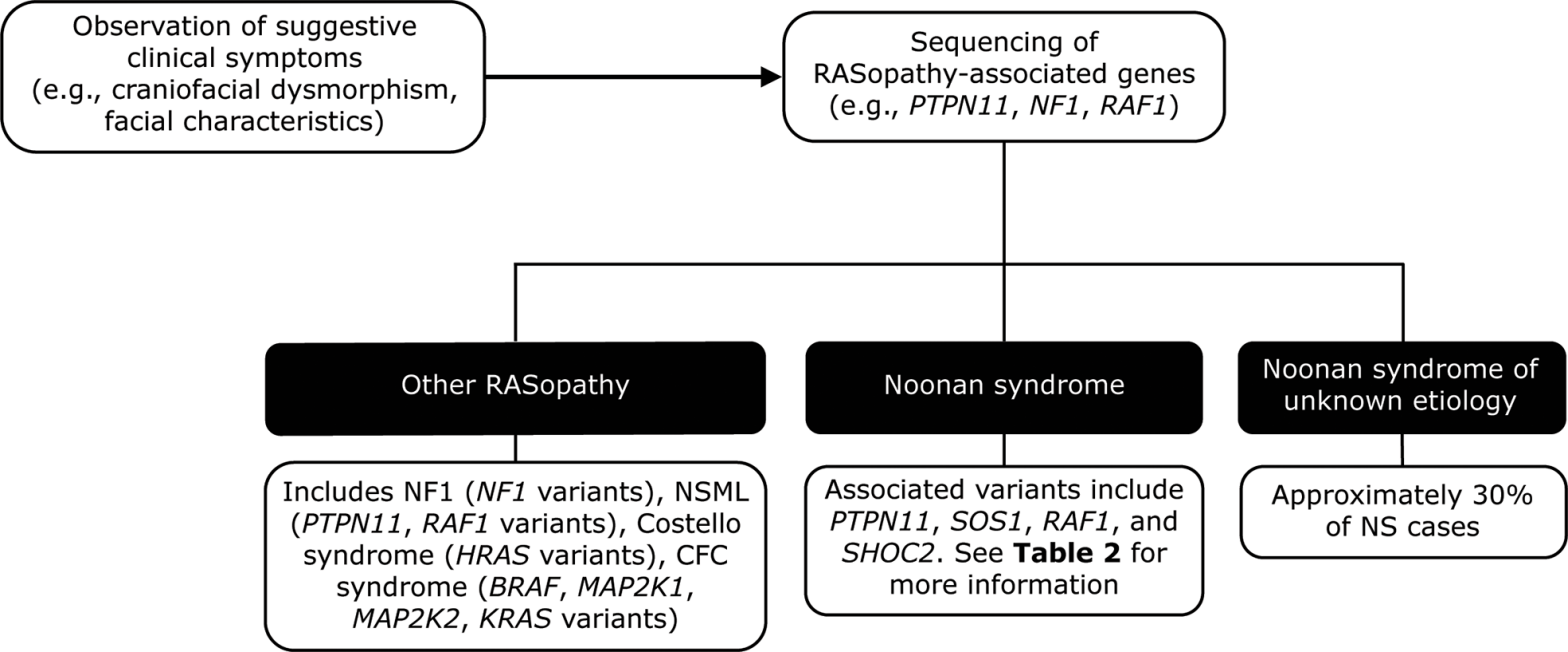
Genetic testing flowchart for NS. NS is typically diagnosed based on the observation of features such as craniofacial dysmorphism and facial characteristics. Other RASopathies, including NF1 and Costello syndrome, exhibit similar phenotypes to that of NS and can be distinguished using genetic testing approaches. However, a limitation of genetic testing is that approximately 30% of NS cases are not attributable to a known gene variant. BRAF, V-Raf murine sarcoma viral oncogene homolog B1; CFC, cardiofaciocutaneous; HRAS, Harvey rat sarcoma viral oncogene homolog; KRAS, Kirsten rat sarcoma viral oncogene homolog; MAP2K1, mitogen-activated protein kinase kinase 1; MAP2K2, mitogen-activated protein kinase kinase 2; NF1, neurofibromatosis type 1; NS, Noonan syndrome; NSML, Noonan syndrome with multiple lentigines; PTPN11, protein-tyrosine phosphatase, non-receptor type 11, RAF1, v-raf-1 murine leukemia viral oncogene homolog 1; SHOC2, SHOC2 leucine-rich repeat scaffold protein; SOS1, Son of Sevenless homolog 1.

The diagnosis can be corroborated using fetal ultrasound and echocardiography techniques (69) and confirmed by prenatal and/or postnatal molecular testing (see below). After birth, NS may be suspected based on characteristic facial features or the presence of bleeding or cardiac abnormalities (**Figure 2**) (3). Scoring systems have also been proposed to aid in the clinical diagnosis of NS (10). Diagnoses based on clinical features can be confirmed by genetic testing in up to 70% of cases (3).

Molecular genetic testing has identified at least 13 genes with pathogenic variants that contribute to NS (**Table 2**) (63). Genetic testing approaches can include the use of a multigene panel, serial single gene testing, and/or more comprehensive genomic testing with deletion/duplication analysis (1). In general, a multigene panel is the test of choice. If that is not feasible, serial single gene testing beginning with *PTPN11* could be considered, because 50% of individuals with NS display *PTPN11* variants. If either of these approaches fails to confirm a diagnosis in an individual with NS features, more comprehensive genomic testing may be considered when available. However, a limitation of genetic testing is that current technologies reveal no specific molecular defect in roughly 30% of NS cases. Availability of and approval (from both families and insurance carriers) for such testing can also be a barrier to diagnosis.

The characteristic features of NS are often similar to those of Turner syndrome (TS) (discussed in more detail below). However, a few key features distinguish these two syndromes. Most notably, TS only affects females, while NS affects males and females equally (4). Additionally, individuals with TS often display primary ovarian insufficiency, while fertility does not seem to be affected in girls with NS. Finally, NS is typically caused by a RAS/MAPK pathway gene variant, while TS is characterized by the complete or partial loss of the second X chromosome in females (1).

Several other syndromes, including cardiofaciocutaneous (CFC) syndrome, NS with multiple lentigines, neurofibromatosis type 1 (NF1), and Costello syndrome also show significant phenotypic similarities to NS (1). These syndromes all result from RAS/MAPK signaling pathway variants and are classified as RASopathies. Many can be distinguished based on their clinical features. For example, individuals with CFC syndrome tend to have more severe facial features, feeding difficulties, and intellectual difficulties than those with NS. Costello syndrome can be distinguished by its associated hair loss, moderate intellectual difficulties, and increased pigmentation that develops with age (1). Several syndromes can be genetically differentiated from NS (2); for example, Costello syndrome is caused by *HRAS* variants not observed in NS (1), while NF1 is characterized by variants in the neurofibromin 1 (*NF1*) gene (5). A combined neurofibromatosis-Noonan syndrome, in which affected individuals display symptoms of both NF1 (especially café-au-lait spots) and NS, has also been observed. Genetic studies have revealed that neurofibromatosis-Noonan syndrome may result from specific *NF1* variants, including the p.R1809C variant or in rare instances from co-occurring *NF1* and *PTPN11* variants (99–101).

Because NS is such a genetically and phenotypically heterogeneous disease, its identification, diagnosis, and management can be challenging. Key advances in improving the diagnosis and care of individuals with NS include the establishment of diagnostic and management guidelines and patient support groups. The current published guidelines include those from: DYSCERNE Noonan Syndrome Guideline Development Group^1^, Romano and colleagues (1), National Organization for Rare Disorders (NORD)^2^, and BMJ Best Practice^3^. Despite their limitations (all lack a standardized consensus development process), they have been clinically useful and have illustrated the need for further studies, molecular diagnoses, and data collection. Patient support groups for individuals with NS include the Noonan Syndrome Foundation, RASopathies Network, NORD, the Human Growth Foundation, and the Magic Foundation. Improved education of providers (particularly pediatricians) is critical, as patients with NS will need referral to multiple care specialists to address the many comorbidities that accompany the disorder.

### Treatment

Appropriate evaluations should be conducted at the time of diagnosis, as recommended in previous guidelines, to identify associated comorbidities (1, 2). Treatment of most NS-associated comorbidities does not differ from treatment of the same conditions in unaffected individuals. Treatments for the more common NS abnormalities are described below and include some NS-specific treatment considerations and differences in approach.

#### Growth/puberty

Children with NS and growth failure that cannot be explained by another comorbidity should have their nutrition optimized and baseline laboratory assessments performed. Puberty is often delayed in individuals with NS and may need to be induced, but induction timing should be carefully considered to avoid negatively impacting height outcomes (1, 62). Patients should be referred to a pediatric endocrinologist for management of pubertal issues and growth failure and consideration for GH therapy. GH therapy has been approved by the Food and Drug Administration (FDA) for use in NS since 2007 and is now licensed for use in Switzerland, South Korea, Israel, Brazil, Japan, and Europe (2).

Early initiation of GH therapy for NS-associated short stature can lead to significant short- and long-term increases in height velocity, height standard deviation score (SDS), and adult height (**Table 3** and **Figures 1A, B**) (45, 62, 102, 110–112). Earlier initiation, longer duration of therapy, and increased height SDS at the time of treatment initiation are all positive predictors of response to GH treatment (110–113). Additionally, there is no difference in response to GH therapy between those who are GH-deficient and those who are GH-sufficient (110, 114). IGF-I concentrations are low in patients with NS but increase upon initiation of GH treatment. It is suggested that IGF-I levels be carefully monitored during GH dose escalation and maintained between 0 and +2 SDS (46).

**Table 3:** Impact of GH on height in Noonan, Turner, and Prader-Willi syndromes.

	Noonan	Turner	Prader-Willi
**FDA-approved GH dose**	≤66 mcg/kg/d	47-67 mcg/kg/d	34-50 mcg/kg/d*^,†^
**FDA approval year**	2007	1996	2000
**Average untreated adult height**	163 cm (males) 153 cm (females)	143 cm	155 cm (males) 147 cm (females)
**GH-treated adult height**	Improves final height by 9-13 cm	Improves final height by 5-8 cm	Improves final height by 18 cm (females), 20 cm (males)
**Recommended age at treatment initiation**	Before puberty^‡^	Recommended by 4-6 years, as early as 9 months	Approved for individuals ≥2 years old, recommended as early as 4-6 months
**References**	(1, 102, 103)	(25, 104–107)	(33, 39, 108, 109)

*It is recommended that GH dosing in obese patients be based on the ideal (nonobese) body weight (108). ^†^Up to 1 mg/m^2^/day in children, with subsequent dose adjustments based on growth velocity, head circumference, and serum IGF-I. ^‡^Some evidence demonstrates that height SDS at the time of puberty is correlated with near-adult height (110). Therefore, beginning GH treatment when growth failure is first identified (so that height is normalized by pubertal onset) may be warranted.

FDA, Food and Drug Administration; GH, growth hormone; SDS, standard deviation score.

Despite initial concerns regarding GH resistance or poor response in patients with *PTPN11* variants, longer-term studies have shown similar growth responses and adult height outcomes in individuals with and without *PTPN11* variants (9, 103). Further studies are needed to clarify the role of the different RAS/MAPK pathway aberrations in GH responsiveness.

Several concerns have been raised about the safety of GH in individuals with NS, particularly in those with cardiac-related comorbidities. However, multiple studies have revealed no changes in cardiac parameters, including left ventricular wall thickness, and few cardiac-related adverse events in GH-treated individuals with NS (1, 45, 46, 62, 112). Further studies are needed to substantiate the continued safety of GH use in NS with respect to cardiac risks. At this time, for individuals with a clinical diagnosis of NS for whom GH therapy is indicated and who have cardiomyopathy, baseline and regular ultrasound examinations should be performed. Those with severe cardiomyopathy should not be treated with GH (115).

Because of an increased risk of cancer in individuals with NS, there are specific risk considerations for all patients with a clinical diagnosis of NS for whom GH therapy is indicated. Patients and caregivers should be informed that the risk of developing cancer in association with GH treatment has not been adequately studied (116). Studies reporting increased malignancy rates with GH treatment are sparse and tend to include only small patient cohorts (62). Primary brain tumors appear to be increased in individuals with NS (92) and therefore one group has suggested performing brain magnetic resonance imaging (MRI) screens prior to beginning GH therapy (91, 117).

Prior to therapy initiation, a molecular test should be performed to identify pathogenic variants. For those with gene variants associated with a high risk of myeloproliferative disorders, the GH treatment decision should be carefully discussed with parents and either deferred until after the age of 5 years or begun with appropriate surveillance prior to 5 years of age (118, 119).

#### Cardiovascular

When NS is first diagnosed, an electrocardiogram and an echocardiogram are recommended to diagnose cardiac defects and CHD as early as possible (16). Treatment for cardiac-related conditions tends to be the same as would be used for the general population (1). Importantly, PS treated with percutaneous valvuloplasty has a higher reintervention rate in individuals with NS than in those without due to the dysplastic pulmonary valve observed in NS (16). In these cases, surgical valvotomy, which seems to be equally efficacious in individuals with and without NS, may be required (16). Additionally, there is a poor prognosis associated with HCM in infants presenting with congestive heart failure before the age of 6 months (31% 1-year survival) (120). Recently, inhibition of mitogen-activated protein kinase kinase, or MEK, was reported to reverse this progressive myocardial hypertrophy in two infants (121).

#### Coagulopathies/malignancy

Because individuals with NS can experience multiple bleeding disorders, clinical evaluations should be performed at diagnosis and hemostatic coverage in symptomatic individuals must be considered prior to any surgery (122). Treatment for bleeding issues in NS must be based on the specific hematologic anomalies identified in the individual. Aspirin and aspirin-containing medications should be avoided (1).

For individuals with NS who display specific variants (primarily codon 61 and p.T731 *PTPN11* variants or p.T581 *KRAS* variants) that confer a high risk of myeloproliferative disorders/juvenile myelomonocytic leukemia, surveillance *via* yearly physical examination is recommended between birth and 5 years of age. An assessment of spleen size and a complete blood count with differential should be performed every 3 to 6 months (118). For all other individuals with NS and non–high-risk or unknown gene variants, no routine surveillance has been recommended, but practitioners should maintain an increased awareness and low threshold for investigating new potential tumor-related symptoms, including new-onset seizures, headaches, and petechiae (118).

#### Genitourinary

A baseline kidney ultrasound should be performed at diagnosis. Orchiopexy should be performed by the age of 1 year if testicles remain undescended at that time (2).

#### Cognitive/developmental

Annual developmental screening should be conducted with neuropsychological testing if screening results are abnormal. Early intervention programs and individualized education strategies should assess and address issues. Special education, speech therapy, physical therapy, and/or occupational therapy may be required in patients with learning issues, language difficulties, and difficulties with gross and fine motor skills (1, 12). Consistent evaluation of hearing loss is necessary to improve hearing management and to reduce its effects on children with developmental disabilities (60).

### Transition and multidisciplinary care

NS is a genetically and phenotypically heterogeneous condition. Therefore, an accurate diagnosis and appropriate care can be difficult to obtain, particularly during the transition period between pediatric and adult health care systems. Most of the comorbidities of NS that occur during childhood require follow-up through adulthood. These include, but are not limited to, cardiac and musculoskeletal defects; bleeding disorders and tumor predisposition; lymphatic problems; and cognitive, behavioral, and mental health issues (2).

It is particularly important to address the cardiac issues that arise in adults with NS. Although cardiac interventions may be undertaken during pediatric care, many individuals will require additional interventions in adulthood. One-third of adults with NS have ongoing cardiac issues that require the use of pacemakers, defibrillators, or drugs for heart failure or arrhythmias (2). Adult CHD centers and guidelines published by the American Heart Association provide best practices for managing the transition of adolescents with CHD to adulthood (123).

Clinical neuropsychological evaluation of the cognitive, adaptive, and psychological domains of functioning should extend from childhood through the transition phase in individuals with NS (12). Adults with NS may also experience social and interpersonal issues, which can lead to anxiety, depression, and lower overall life satisfaction (1, 17). Ideally, a multidisciplinary team composed of geneticists, cardiologists, endocrinologists, and behavioral and mental health experts will be involved in a coordinated transition. Management guidelines, the information provided here, and involvement with patient support groups may also help during the transition and through adulthood.

## Turner syndrome

TS results from complete or partial loss of the second X chromosome in females (106). The prevalence of TS is 1 in every 2000 to 2500 live female births (106). Individuals with TS display an array of clinical features including short stature, primary ovarian failure, cardiac anomalies, and neurodevelopmental difficulties. Multinational consensus guidelines for the diagnosis and management of women with TS have been published (25).

### Features

Clinical presentation of TS can be highly variable depending on age and whether a patient displays complete versus partial X-chromosome loss or has 45,X monosomy versus mosaicism. Prenatal findings may include nuchal translucency, cystic hygroma, left-sided obstructive cardiac anomalies, or renal abnormalities. These features are not diagnostic for TS but are highly suggestive and mandate postnatal karyotyping, chromosomal microarray, and/or sequencing-based analysis (106, 124). Other symptoms observed in infancy, including webbed neck, lymphedema, and coarctation of the aorta, also indicate a requirement for genetic testing (27). Characteristic features observed in individuals with TS can be found in **Table 1**.

**Figure 3:**
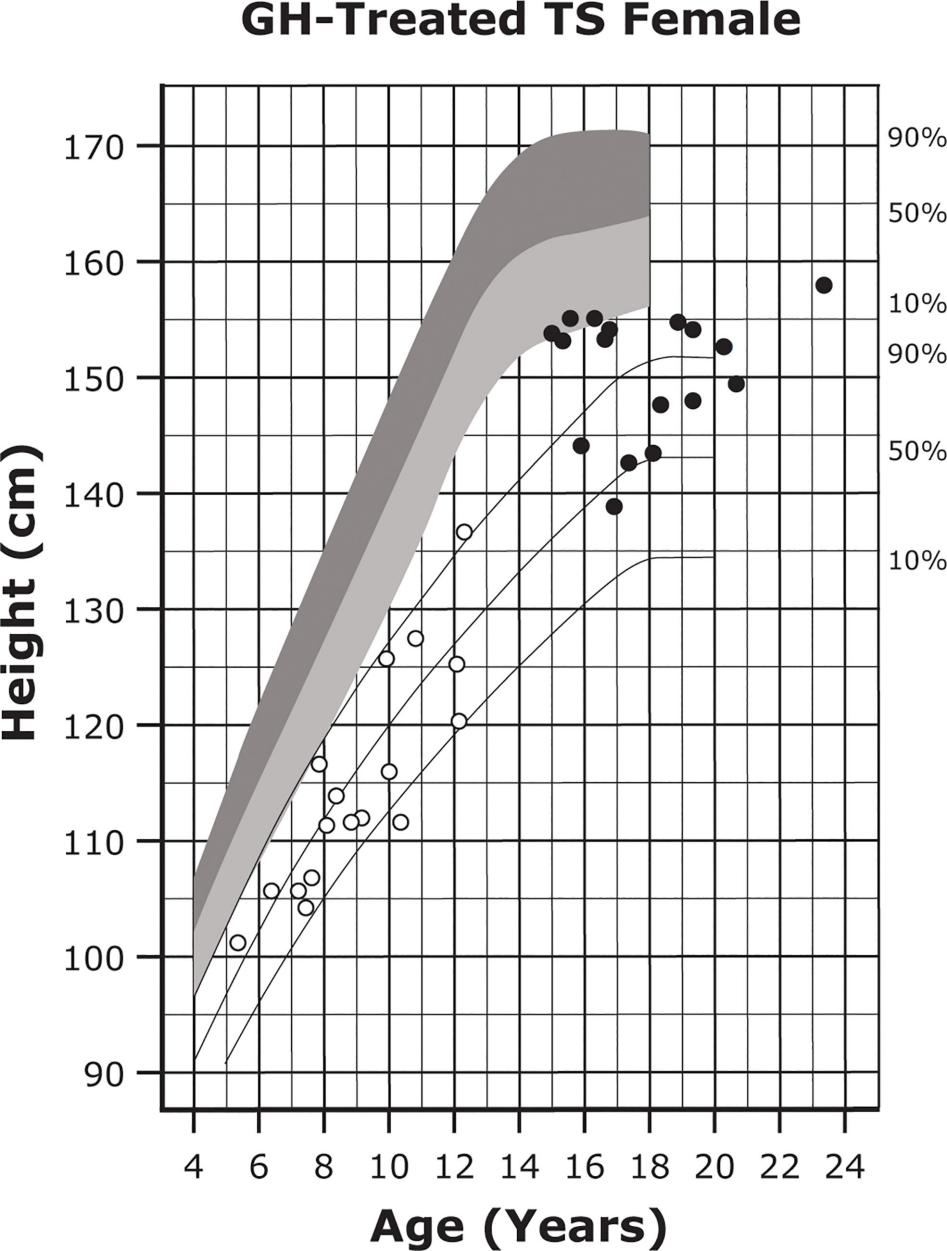
Growth curves for patients with TS. Heights of individuals with TS at the start of GH treatment (open symbols) and at final height (solid symbols). Heights from a reference TS population are shown with solid lines and overlaid on normative growth curves from the National Center for Health Statistics (shaded area), with normative 50th to 90th percentiles in dark shading and normative 10th to 50th percentiles in light shading. Reprinted from J Pediatr, 132(2), Rosenfeld RG, et al. 319-24, ^©^1998 Mosby, Inc. with permission from Elsevier. GH, growth hormone; TS, Turner syndrome.

Up to 95% of individuals with TS exhibit short stature (24). Approximately two-thirds of the height deficit can be attributed to haploinsufficiency of the short stature homeobox (*SHOX*) gene, which encodes a transcription factor mainly expressed by osteogenic cells (24, 125). Left untreated, individuals with TS achieve adult heights of approximately 143 cm (~20 cm shorter than the average for the general female population) (**Figure 3**) (24, 104, 105). It has been suggested that growth deficit severity is related to X-chromosome haploinsufficiency and possibly to genomic imprinting impacted by the parental origin of the missing X chromosome, although these findings have been inconsistent across studies (24, 106).

While individuals with TS display some similar characteristics to those with NS, particularly in the fetal and neonatal periods, TS affects only females and has an entirely different underlying genetic basis. Further, individuals with TS present with more left-sided cardiac defects and an increased prevalence of renal abnormalities. Finally, individuals with TS tend to exhibit primary hypogonadism which causes both amenorrhea and infertility.

### Other comorbidities

Besides short stature, TS is accompanied by several comorbidities including abnormalities of the cardiovascular, metabolic, gastrointestinal, genitourinary, and nervous systems. Some groups have attributed the diversity of phenotypic features seen in individuals with TS to the parental origin of the X chromosome. Individual reports have indicated a parent-specific effect of X-chromosome inheritance on growth outcomes, altered lipid profiles, renal abnormalities, cardiovascular abnormalities, or cognitive functioning; however, other studies have failed to consistently reproduce these findings (21, 126–130). As a result, genetic testing to determine the X chromosome parent-of-origin in individuals with TS is not currently recommended.

#### Cardiovascular

Cardiac defects (**Table 1**) are seen in approximately 50% of individuals with TS and may contribute to early morbidity (25, 106, 131–133). An elongated transverse aortic arch, observed in nearly 50% of cases, may not always be clinically relevant on its own but may reflect abnormalities in the aortic wall, indicating an increased risk for complications such as aortic dilation and subsequent dissection (20). In general, left-sided cardiac defects are associated with an increased risk of aortic dissection, aneurysm, and sudden death (23, 134). The proposed mechanism underlying aortic aneurysm in individuals with TS is the increased synthesis of matrix metalloproteinases (and decreased synthesis of their inhibitors), which degrade collagen and elastin, thereby thinning and weakening the aortic wall (22, 134). Bicuspid aortic valve occurs 30 to 60 times more frequently in individuals with TS than in the overall female population and may be an independent predictor of TS (25). Although cardiac problems are seen in all individuals with TS, they may be more severe in association with the 45,X karyotype or specific copy number or X chromosome variants (135, 136).

#### Puberty

TS is associated with pubertal delay and ovarian insufficiency (**Table 1**) (106, 137). While spontaneous puberty has been reported in up to one-third of individuals with X-chromosome mosaicism, most females with TS will require pubertal induction with replacement sex hormones (23, 106).

#### Renal

Congenital renal abnormalities (**Table 1**) occur about nine times more frequently in individuals with TS than in the general population (27, 106, 138).

#### Metabolic

Many individuals exhibit metabolic issues, including obesity, glucose intolerance, and type 2 diabetes (26, 106, 139). Metabolic factors, including perihepatic and pericardial fat thickness, may also predict increased risk for cardiac problems in individuals with TS (140).

#### Cognitive/developmental

Although individuals with TS typically have a normal IQ, about 10% present with an intellectual disability (106, 141, 142). Girls with TS tend to have challenges with executive functioning and motor coordination; ASD and ADHD are also common (106, 142, 143). Individuals with TS tend to have average to above-average reading and verbal abilities but fall short of age-appropriate averages in overall comprehension and visuospatial skills, including mathematics and processing speed (142, 144). Individuals also show impaired social functioning, specifically in the areas of facial recognition, fear processing, and interpreting social cues (141, 142). These findings may possibly be attributed to abnormal structural and connective differences in the brains of individuals with TS (145–148). It has also been suggested that differences in X-linked parent-of-origin and imprinting effects influence neurocognitive outcomes in patients with TS, although additional studies are needed (149–151).

#### Auditory

Ear and hearing issues are present in about 19% of individuals with TS and include external ear abnormalities, frequent otitis media, cholesteatoma, and conductive or sensorineural (in adults) hearing loss (27, 106, 152). Ear and hearing issues may require the placement of myringotomy tubes (25).

#### Oncologic

Individuals with TS also appear to be at a slightly increased risk for the development of certain neoplasms (e.g., meningiomas, melanomas), perhaps due to an increased frequency of copy number variants, autosomal aneuploidies, and chromosomal instabilities (25, 128, 135). Some individuals (approximately 10% of cases) with TS harbor Y chromosome sequences; these individuals display an increased prevalence of gonadoblastoma and germ cell tumors. The presence of Y chromosome genetic material is a consideration for gonadectomy (23, 25).

#### Autoimmune

Individuals with TS may be up to twice as likely as the general female population to develop certain autoimmune disorders, including Hashimoto thyroiditis, vitiligo, juvenile idiopathic arthritis, celiac disease, and inflammatory bowel disease (27, 106, 153). The mechanisms underlying the development of these diseases remain unclear but may be related to X-chromosome haploinsufficiency (153, 154).

### Diagnosis

The genetics underlying TS encompass several different X-chromosome anomalies, including 45,X (55%) and X-chromosome mosaicism (25%). About 20% of individuals with TS will have a more complex karyotype, including deletion of the short arms of the X chromosome or ring X chromosome (155). About 75% of all cases are thought to originate with the sperm missing an X chromosome (127, 156).

**Figure 4:**
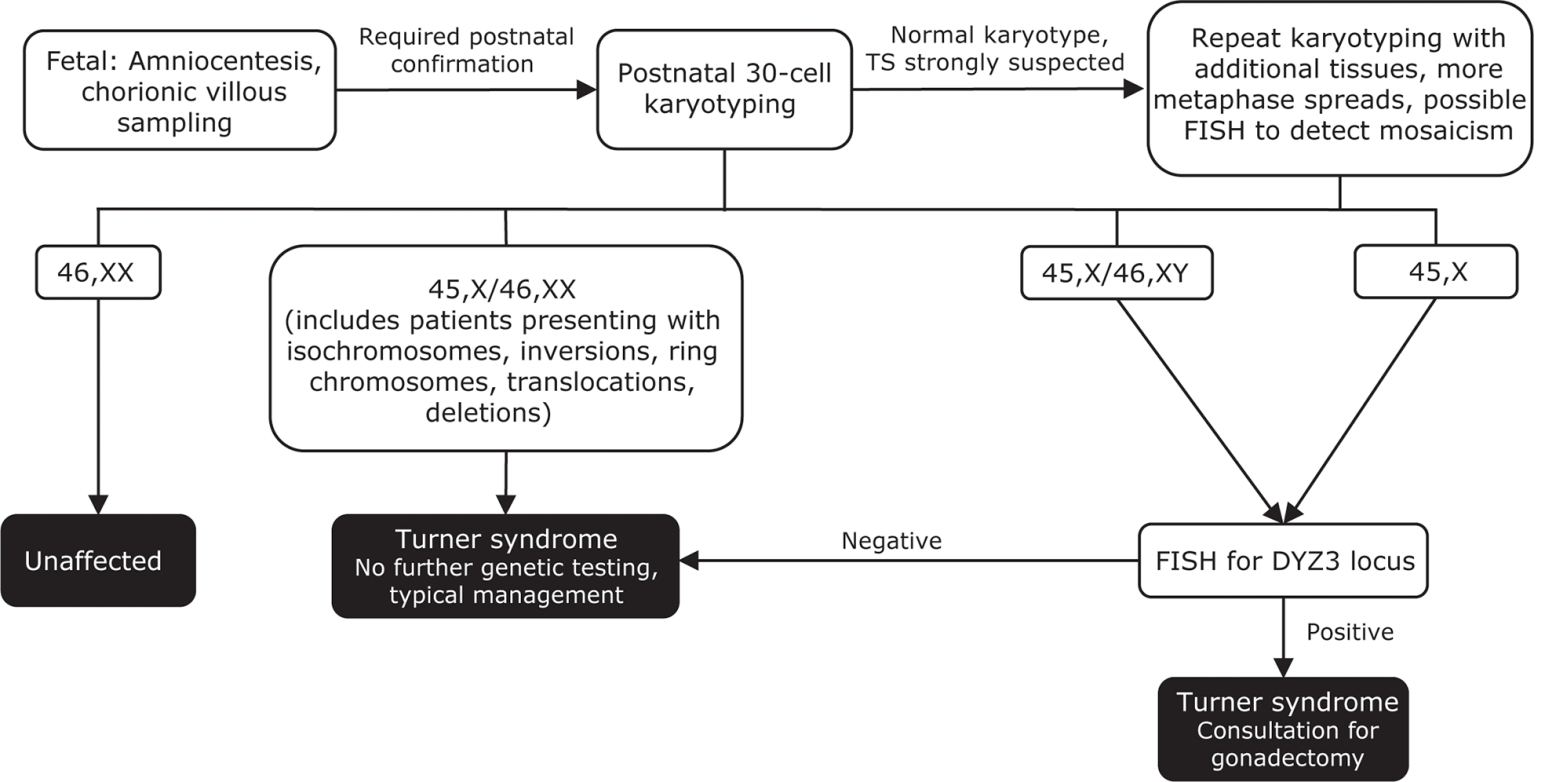
Genetic testing flowchart for TS. A diagnosis of TS can be suspected based on fetal tests, including amniocentesis and chorionic villous sampling. However, all diagnoses require postnatal confirmation *via* 30-cell karyotyping to identify defects such as a missing X chromosome, the presence of a Y chromosome, or other X chromosome problems (eg, deletions, ring chromosomes, isochromosomes, translocations). In cases where TS is strongly suspected based on clinical features but the first karyotype comes back normal, additional karyotyping should be performed using additional tissues and/or more metaphase spreads. FISH can also be used to detect X-chromosome mosaicism. Cases that present as 45,X/46,XY and 45,X should undergo additional FISH for the DYZ3 locus, which will detect the presence of Y-chromosome material. If Y-chromosome material is present, the risk for gonadoblastoma is elevated and patients should be referred to a specialist for consultation for gonadectomy. Adapted from Ackermann A, Bamba V. J Clin Transl Endocrinol 2014;1:61-5. FISH, fluorescence in situ hybridization; TS, Turner syndrome.

TS should be suspected in any female infant who presents with suggestive fetal or postnatal features (141). Amniocentesis or chorionic villous sampling may reveal TS in a fetus (**Figure 4**) (25). Noninvasive prenatal testing of maternal cell-free DNA is currently not routinely recommended, as it does not adequately predict TS or detect mosaicism (157, 158). Regardless of the prenatal testing performed, postnatal karyotyping is always required to confirm a diagnosis of TS (25, 106).

Other clinical characteristics that should prompt testing for TS include short stature, left-sided obstructive cardiac abnormalities, renal anomalies, multiple nevi, frequent bouts of otitis media, hearing loss, TS-specific neuropsychological issues, and delayed sexual development (25, 106, 156).

Despite the variability of phenotypes among individuals with TS, standard karyotyping can detect TS with up to 95% confidence (25, 159). In cases where TS is strongly suspected but the result of the first karyotype is normal, additional tissues or more metaphase spreads may need to be analyzed or chromosomal microarrays used to detect either mosaicism or complete chromosome loss (106, 141).

Early diagnosis is key; patient outcomes are improved with timely initiation of care. In particular, earlier initiation of GH may result in increased height velocity and adult height outcomes or may narrow the height gap at an earlier age (25, 27, 160). Studies have revealed that up to 30% of TS diagnoses may not be made until adulthood (161). Additionally, short stature alone often does not prompt appropriate testing for genetic disorders in girls. However, improved diagnostic guidelines and screening procedures are decreasing this diagnostic delay (27, 106, 161). Education of providers on the clinical signs and symptoms of TS may help to ensure early diagnosis and care for TS-associated comorbidities (25).

It has been proposed that screening for TS be added to NBS programs. However, karyotyping can be expensive, time-consuming, and labor-intensive, as it requires living cells. Pyrosequencing, real-time polymerase chain reaction, and whole exome sequencing, which are DNA-based, have all been proposed as alternative NBS methods (25, 124).

### Treatment

#### Growth

Individuals with TS and short stature can receive GH replacement therapy to promote catch-up growth. Treatment with GH is recommended to start around 4 to 6 years of age (earlier if growth failure is present) and may result in a height SDS gain of up to 1.5 (**Table 3** and **Figure 3**) (25, 162–164). In some instances, individuals who start GH at a younger age may display better height outcomes and improved body composition and bone growth (25, 27, 106, 107, 160, 164). GH treatment is associated with an average height gain of 1 cm per year based on randomized studies, and improved height gain has been reported in some observational trials (25, 104, 165, 166).

Once GH treatment is started, IGF-I concentrations should be monitored at least annually and should ideally be maintained at or below an SDS of +2. Upon testing, if the IGF-I SDS is greater than +3, the GH dose should be decreased, and if the SDS is between +2 and +3, the provider should use their best clinical judgment regarding dosing (25). Girls with TS on GH therapy are at increased risk for intracranial hypertension, slipped capital femoral epiphysis, and worsening of scoliosis, presumably due to accelerated growth rates (25, 106).

Individuals with TS are at a higher risk of metabolic disorders associated with insulin resistance. These disorders may be transiently exacerbated during GH treatment with resolution after treatment completion (141). Plasma glucose and hemoglobin A1c should be measured annually beginning around 10 years of age. Girls with TS should also receive counseling to highlight the importance of a healthy diet and physical activity (26, 106).

In addition to GH, non-aromatizable androgens, such as oxandrolone, have been used to stimulate height velocity in individuals with a late diagnosis of TS or with concern for extremely short height outcome (163), although this practice is more common in Europe than in the US. Current guidelines suggest adding treatment with oxandrolone at ≥10 years of age (25). Delayed breast development, mild excess body hair, and voice deepening have been noted in some oxandrolone-treated patients, emphasizing the need to delay treatment until later in childhood and to keep doses below 50 mcg/kg/d (25).

#### Puberty

Many individuals with TS will require treatment with estrogen and progestin to induce breast development and menstruation. Transdermal estradiol, which is associated with decreased thromboembolic and carcinogenic risk relative to other routes of administration, is the preferred starting method and should be initiated at a low dose (3-7 µg/day) at about 11 years of age and slowly increased to the full adult dose (25-100 µg/day) over the course of approximately 2 years (23, 25, 167, 168). Doses should be increased by between 25% and 100% every six months to achieve the full adult dose in the recommended time frame (25). Estrogen treatment also has beneficial effects on bone mineral density, fasting glucose, and cholesterol levels (106, 139). Progestin should be started after about 2 years of estrogen therapy or once breakthrough bleeding has occurred (25, 106).

Some studies have suggested that induction of puberty can promote earlier epiphyseal closure, thereby compromising adult height outcomes (169). However, the results of many of these studies are underpowered and inconclusive. Therefore, timing of pubertal induction should be an individual decision made in careful consultation with providers who have experience treating TS and considering growth potential, bone age, and psychosocial aspects of undue pubertal delay.

Levels of follicle-stimulating hormone and luteinizing hormone should be evaluated before beginning pubertal induction to confirm the presence of hypergonadotropic hypogonadism (106). Individuals with TS and normal ovarian function should receive counseling about their options for fertility preservation as early as possible (106).

#### Cardiovascular

Cardiac abnormalities are detected using echocardiography, MRI, or magnetic resonance angiography (MRA) (23, 133, 170). Some abnormalities, including an elongated transverse aortic arch and partial anomalous pulmonary venous return, can only be observed *via* MRI/MRA. Current guidelines recommend echocardiographic evaluation of newly diagnosed girls with TS irrespective of their cardiac risk status and regular monitoring thereafter to detect arrhythmias (25, 106). MRI/MRA should be considered as soon as the procedure can be performed without sedation; however, implementation can be limited by difficulties in obtaining insurance approval (20). Vascular issues, such as coronary artery disease, are also common among individuals with TS and can be detected using computed tomography angiography or cardiac MRI (133, 170).

Treatment for cardiac issues depends on the severity of the abnormality and on other associated risk factors (e.g., obesity, hypertension). Most individuals are stratified by their overall cardiac risk level and screened at regular intervals per the current consensus guidelines (25, 136). It is recommended that girls in the high-risk category avoid highly competitive sports and intense weight training (106). For cardiac abnormalities such as aortopathies and left-sided obstructive lesions, cardiac surgery is usually indicated (25, 171). Individuals with TS who undergo cardiac surgery experience increased morbidity and lengthier hospital stays but do not display increased peri-operative mortality relative to patients without TS (171). Pregnancy is associated with an increased risk of cardiac-related death due to aortic dissection in individuals with TS; therefore, alternatives including adoption and surrogacy should be considered, even if fertility indices are normal. Assisted fertility should be undertaken with caution in a center experienced in this population. If pregnancy does occur (either spontaneously or through *in vitro* fertilization), regular cardiac assessments, including echocardiography, electrocardiography, and MRI, should be performed throughout the pregnancy and the postpartum period (20, 23). Implantation of multiple fertilized embryos may result in multiple gestation with an increased risk of cardiac complications (23).

#### Neurologic/cognitive

To ensure prompt recognition and treatment of TS-associated neurocognitive deficits, some publications suggest yearly neurodevelopmental evaluation for at least the first three years of life (25, 142). Identification of issues during these screenings can lead to prompt intervention and improved outcomes. For example, cognitive behavioral therapy and classroom modifications can help improve learning outcomes related to executive functioning (106). ADHD medications can also have a positive effect on academic achievement; however, medical history and cardiac health should be considered before initiating treatment with stimulant medications (106, 142). Counseling should also be made available to individuals with TS, as they frequently experience low self-esteem, anxiety, and depression (106, 142).

### Transition and multidisciplinary care

Appropriate transitioning of individuals with TS from pediatric to adult health care providers is critical to ensure continuity of care. Individuals with TS tend to visit their providers with decreasing frequency as they age (typically due to competing social, educational, and financial priorities) and may be lost to follow-up in their adult years. These individuals therefore do not receive the recommended screening and care for disease-associated comorbidities, leading to poor health outcomes (25). Notably, adequate medical care following the transition occurs in only 3.5% of individuals with TS (172).

The Transition Readiness Assessment Questionnaire (TRAQ) 5.0, a self-assessment that examines patients’ health knowledge and literacy (including managing daily activities, keeping appointments, and managing medications), may be an important tool for providers to assess an individual’s readiness prior to their transition to adult health care (25, 172). A comparative study assessing individuals with TS and individuals without a chronic condition revealed that participants with TS display lower TRAQ scores than their unaffected peers and may require additional support during the transition process (173).

Current guidelines for the management of TS recommend a planned and staged process for the transition from pediatric to adult health care systems. Coordination between pediatric and adult providers is required to ensure continuity of care. It is recommended that providers use available transitional tools, including the TS Pediatric to Adult Care Transition Toolkit^4^, to support these individuals and to help them identify the role of each member of their care team (25).

The importance of multidisciplinary treatment extending from childhood through adulthood cannot be overemphasized. Clinics, patient support groups, and even telemedicine health visits may help to guide individuals with TS as they make healthy lifestyle choices related to diet, exercise, and fertility planning (25). Dedicated multidisciplinary clinics aimed at supporting individuals with TS during and after the transitional period can also increase identification and treatment of comorbidities in adults (25, 174). A multidisciplinary care team for adults with TS will ideally include specialists in genetics, cardiology, reproductive endocrinology, audiology, and psychology/neuropsychology. Multidisciplinary care and regular monitoring are especially important in pregnant women with TS, as pregnancy is an additional risk factor for cardiac issues such as heart disease and aortopathy (23, 25, 106). Comprehensive care of individuals with TS from childhood to adulthood will ensure that they achieve their full potential and optimal quality of life.

## Prader-Willi syndrome

Prader-Willi syndrome (PWS) is a rare genetic disorder that occurs in approximately 1 in every 10,000 to 20,000 individuals (31, 35, 36, 38, 39). The syndrome affects males and females equally (175) and is the most common known genetic cause of obesity (29, 35, 36, 39). The disorder is characterized by loss of paternally expressed genes in the imprinted region of chromosome 15q11-q13. Mechanistically, this can result from deletions within the paternal chromosome 15, maternal uniparental disomy 15 (UPD 15; inheritance of both copies of chromosome 15 from the mother and none from the father), or (infrequently) imprinting defects (38, 39, 176–180).

### Features

Clinically, infants with PWS are characterized by severe hypotonia and failure to thrive due to feeding difficulties. *In utero*, they are typically within a normal size range but may exhibit polyhydramnios (181) and decreased movement due to fetal hypotonia (29, 30, 36, 38, 39). In infancy, persistent hypotonia is accompanied by feeding problems, lethargy, and poor suck, often resulting in low body weight (33, 35).

**Figure 5:**
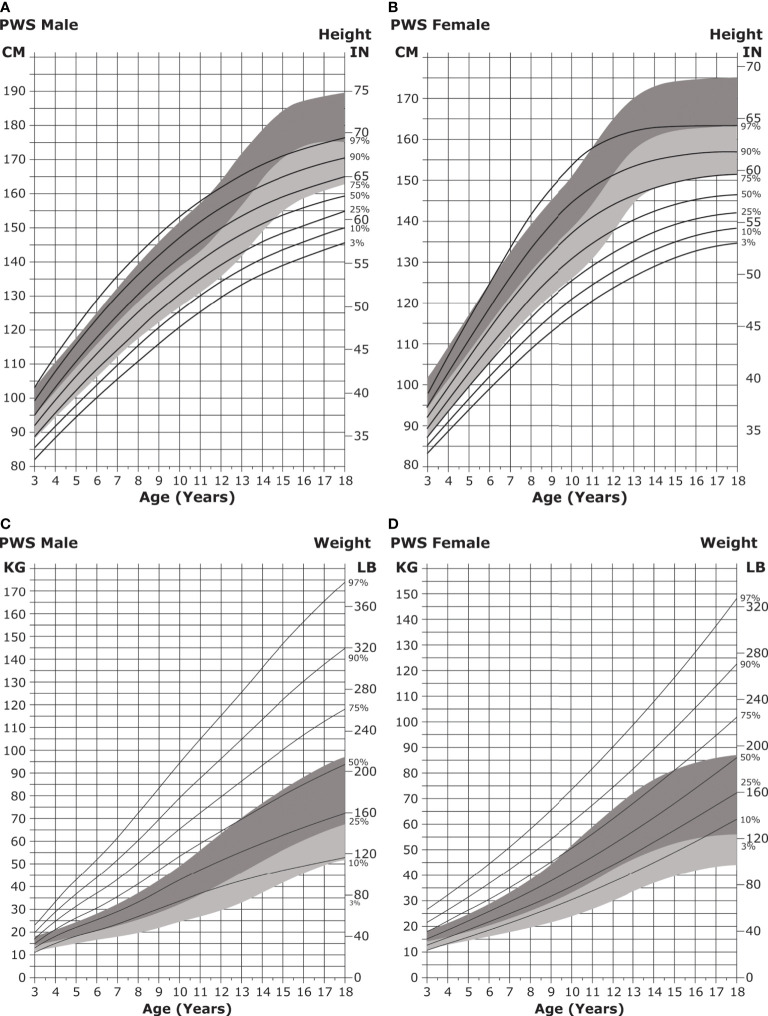
Standardized curves for height and weight of non-GH-treated patients with PWS. **(A, B)** Standardized curves for height of non-GH-treated males **(A)** and females **(B)** with PWS. Heights of individuals with PWS are shown with solid lines and overlaid on the normative percentile ranges (shaded area) with normative 97th to 50th percentiles in dark shading and 50th to 3rd percentiles in light shading. Adapted from Butler MG et al. Pediatrics 2015;135(1):e126-35. **(C, D)** Standardized curves for weight of non-GH-treated males **(C)** and females **(D)** with PWS. Weights of individuals with PWS are shown with solid lines and overlaid on the normative percentile ranges (shaded area) with normative 97th to 50th percentiles in dark shading and 50th to 3rd percentiles in light shading. Adapted from Butler MG et al. Pediatrics 2015;135(1):e126-35. GH, growth hormone; PWS, Prader-Willi syndrome.

Infant length is typically normal in the first few months of life but falls below normative growth within the first year (182). Short stature becomes particularly apparent during puberty, with the median height for individuals with PWS falling below the fifth percentile for unaffected individuals (33) (**Figures 5A, B**).

The feeding difficulties noted in infancy give way to hyperphagia (excessive eating accompanied by lack of satiety) in childhood and can lead to obesity by 2 to 4 years of age if not controlled (**Figures 5C, D**) (30, 31, 175, 183). It is hypothesized that hypothalamic dysfunction, increased production of the hormone ghrelin (which stimulates appetite), and the decreased activity and caloric requirements observed in individuals with PWS lead to the onset of obesity (31, 33, 35, 36, 38, 39, 184).

PWS is characterized by several distinctive facial features, which are listed in **Table 1**. Individuals with PWS also exhibit decreased saliva production, leading to dry mouth, caries, and other dental issues (40, 185).

### Other comorbidities

#### Metabolic/endocrine

Individuals with PWS are prone to hypothyroidism, central adrenal insufficiency, body composition changes, and obesity-associated problems, including type 2 diabetes (38, 39, 186). Individuals with PWS and obesity or diabetes are more likely to experience cardiovascular disease, venous thromboembolism, myocardial infarction, and mortality (187, 188). Individuals with PWS and obesity exhibit increased insulin sensitivity and decreased fasting insulin levels relative to individuals with obesity without PWS, suggesting differences in insulin metabolism and regulation of fat patterning (189). Further, the presence of obesity in individuals with PWS is associated with increased rates of metabolic syndrome (relative to individuals with PWS without obesity), defined by at least three of the following characteristics: high blood glucose, low high-density lipoprotein cholesterol levels, increased waist circumference, high triglyceride levels, and high blood pressure (41).

Hypothalamic insufficiencies can predispose those with PWS to seizures and hypogonadism (36). Seizures occur in 10% to 20% of the PWS population, most often developing before 6 years of age (38, 190). Individuals with PWS caused by a chromosome 15q deletion may be more likely to experience seizures than those with PWS caused by UPD, due to the deletion of a cluster of gamma-aminobutyric acid subunit genes on chromosome 15, which are involved in proper neuronal development and functioning (190).

Central and/or primary hypogonadism and pubertal delay is present in nearly all individuals with PWS and is commonly associated with infertility (38, 39). Hypogonadism typically presents as genital hypoplasia and cryptorchidism (31, 33, 36, 181, 191, 192).

#### Cognitive/developmental

Individuals with PWS may present with significant cognitive impairments, including delayed development of language, cognitive, and motor skills (38); however, most exhibit mild learning and/or intellectual disabilities, with an average IQ score around 65 (30, 33, 39). The severity of these impairments may vary depending on molecular genetic class (**Table 4**). While full-scale IQ scores are low across PWS subtypes, verbal IQ scores are significantly lower in individuals with deletions than in those with UPD 15 (194, 197). Conversely, individuals with UPD 15 are more likely to exhibit signs of ASD or psychotic illness, possibly due to the duplication of several key imprinted and maternally expressed genes in the 15q11-q13 region, including *UBE3A* and *ATP10A* (33, 36, 39, 180, 196, 198). Overall, 12% to 41% of children with PWS have ASD (199).

**Table 4:** Phenotypic correlation with molecular genetic class in PWS.

Molecular	Characteristics
**Genetic Class**
**15q11-q13 deletion**	•More severe verbal and performance IQ impairments •Higher pain threshold •More likely to experience seizures •Worse compulsive and self-injurious behaviors •Increased sleep disturbances •Increased need for special feeding techniques •Hypopigmentation •Lower waist-to-hip ratio, higher hip circumference
**Type I deletion**	•Increased behavioral and compulsive problems •Poorer academic performance •Increased developmental delays •Increased neurologic issues
**Type II deletion**	•Better adaptive behavior and social skills relative to type I deletion or maternal UPD
**Maternal UPD**	•Less likely to have typical facial appearance •Higher verbal IQ •Milder behavioral problems •Fewer compulsive behaviors •More likely to show signs of ASD •Increased psychiatric illness and bipolar disorder •Better visual memory •Increased anxiety

ASD, autism spectrum disorder; PWS, Prader-Willi syndrome; UPD, uniparental disomy.

Sources: (33, 35, 38, 189, 190, 193–196).

Behavioral issues, including frequent temper tantrums, stubbornness, and manipulative behaviors, are seen in up to 90% of individuals with PWS but may vary in type or severity between the PWS molecular genetic classes (38, 193). Many children exhibit obsessive-compulsive symptoms such as repetitive questioning and checking behaviors. Others exhibit compulsive food-seeking, hoarding, or self-harming behaviors, such as skin-picking (39, 195, 196, 200). Many compulsive behaviors and psychological issues appear to be worse in individuals with chromosome 15q deletions versus those with UPD 15 (**Table 4**) (193, 195). Individuals with PWS often prefer solitary activities and may exhibit signs of anxiety and depression (196).

#### Sleep

Many individuals with PWS exhibit sleep issues, including altered sleep patterns and obstructive or central sleep apnea (39, 185, 201). These individuals tend to have decreased oxygen saturation, rapid onset of REM sleep, and increased daytime sleepiness (which may impact behavioral issues), particularly in late childhood and adulthood (30, 38, 202, 203). Obesity is a frequent underlying cause of sleep disturbances; however, individuals with PWS with or without obesity tend to experience sleep problems (201, 204). One explanation for this finding may be the decreased muscle mass and tone seen in PWS, which could lead to respiratory distress or collapse during sleep-associated relaxation (33).

#### Skeletal

Orthopedic problems are also prevalent in individuals with PWS. Approximately 10% and 40% of individuals with PWS will exhibit hip dysplasia and scoliosis, respectively, in infancy and early childhood (38, 39). Scoliosis has been suggested to be a comorbidity for respiratory issues including severe sleep apnea (205). Osteoporosis and osteopenia are also common and can increase the risk for stress fractures (39, 206).

### Diagnosis

PWS should be considered in most cases of severe infant hypotonia, failure to thrive, and poor suck and can be clinically diagnosed using a scoring system developed in the 1990s. This system assigns points to major and minor clinical features and establishes point thresholds for positive diagnoses at various ages (30). However, a molecular test is typically required for diagnostic confirmation, as other genetic disorders, including X-chromosome abnormalities and Angelman syndrome, can cause similar phenotypes to PWS (36, 38, 39, 185, 207). Critically, molecular genetic testing can help to determine the underlying PWS genetic subtype, which is correlated with varying clinical features and severity (**Table 4**) (29, 39, 193, 194).

Approximately 60% of PWS cases are caused by a typical 15q11-q13 deletion (either a larger type I or a smaller type II deletion) in chromosome 15. Type I and type II deletions involve the same distal 15q breakpoint (BP3) but different proximal 15q11-q13 breakpoints (BP1 or BP2) and result in deletions of approximately 6 Mb and 5.5 Mb, respectively (180). Notably, several key neurodevelopmental genes are removed in type I but not in type II deletions; thus, individuals with the type I deletion may exhibit increased developmental delays and neurological issues relative to those with the type II deletion (39, 193). However, individuals with both types of deletions tend to have more severe verbal and performance IQ impairments relative to those with other PWS molecular subtypes (**Table 4**) (185, 193, 197). Approximately 35% of PWS cases are due to maternal UPD 15, while the remaining ~5% of cases are due to imprinting defects or chromosomal translocations or inversions (180). Cases of PWS stemming from deletions and UPD 15 are typically spontaneous and future siblings are unlikely to be affected. However, if PWS is traced back to an inherited imprinting defect or a paternally-inherited imprinting center microdeletion, then the chances of having another child with PWS could be 50% (31, 38).

**Figure 6:**
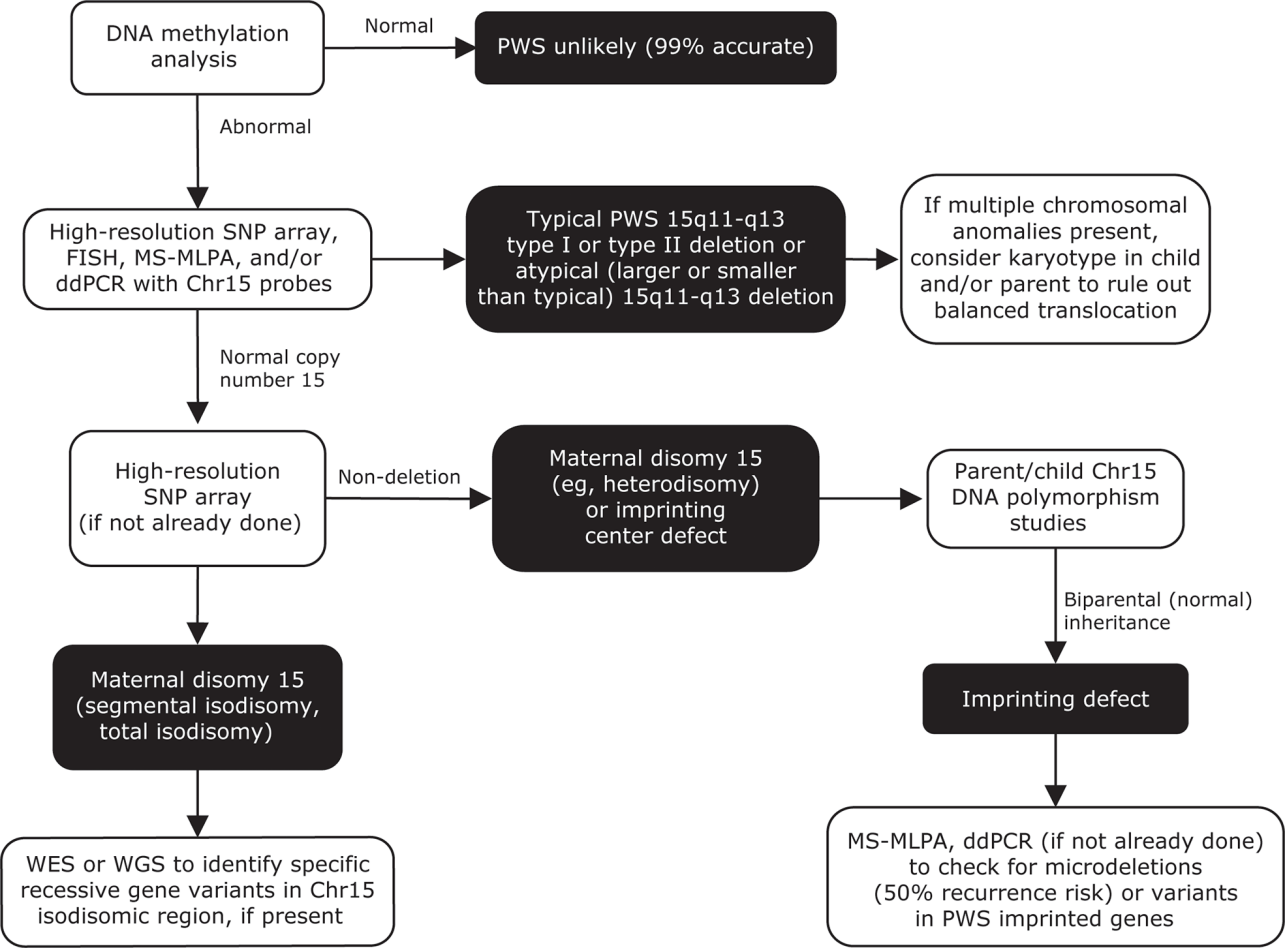
Genetic testing flowchart for PWS. Testing should begin with a DNA methylation analysis, to confirm that only maternally imprinted genes from the 15q11-q13 region are present. High-resolution SNP arrays, FISH, MS-MLPA, and/or ddPCR can be used to detect chromosome 15 deletion subtypes. SNP arrays can also be used to detect maternal UPD 15 subtypes or imprinting center defects. A streamlined approach combining DNA methylation, copy number analysis, and exome sequencing has also been reported for molecular diagnosis of PWS (208). Adapted from Butler MG, Duis J. Front Pediatr 2020;8:154. Chr, chromosome; ddPCR, droplet digital polymerase chain reaction; FISH, fluorescence in situ hybridization; MS-MLPA, methylation-specific multiplex ligation-dependent probe amplification; PWS, Prader-Willi syndrome; SNP, single nucleotide polymorphism; UPD 15, uniparental disomy 15; WES, whole-exome sequencing; WGS, whole-genome sequencing.

A combination of molecular and genetic testing techniques can pinpoint the underlying cause of PWS and 15q11-q13 expression loss (**Figure 6**). Historically, testing began with high-resolution chromosome analysis or, later, DNA methylation analysis, which would confirm that only maternally imprinted genes from the 15q11-q13 region were present (29, 33, 38). Fluorescence *in situ* hybridization could also determine whether a deletion or inversion had occurred in chromosome 15. Currently, high-resolution single-nucleotide polymorphism chromosomal microarrays can be used to identify deletion subtypes (type I or type II) and UPD 15 subclasses (35, 36, 180, 209). These subclasses include segmental isodisomy 15 and total isodisomy 15, which result from varying numbers and sizes of crossover events during maternal meiosis. Individuals with PWS and isodisomy 15 are at risk of secondary genetic conditions if the mother is a carrier of a disorder-associated recessive gene on chromosome 15. DNA polymorphism, methylation-specific multiplex ligation-dependent probe amplification (210), and whole exome sequencing analyses can be used to distinguish between UPD 15 and imprinting defects (211). Recently, a streamlined approach for molecular diagnosis of PWS has been reported that combines DNA methylation, copy number status, and exome sequencing into one assay (208).

Early diagnosis is crucial to minimize many of the comorbidities associated with PWS. Unfortunately, children may be diagnosed anywhere between 18 days and 8 years of age (37, 185), as clinical features can be subtle in presentation and can change dramatically with age (30). In some cases, the need for genetic testing should be immediately evident based on infant hypotonia and failure to thrive with poor suck. However, the lack of rare disease expertise and awareness along with poor accessibility to genetic testing may limit the ability of physicians to order the correct tests and properly diagnose infants (185, 212).

In the US, NBS is regulated at the state level and no states currently test for PWS. However, adoption of molecular testing for PWS in newborns, whether as a part of NBS or due to specific perinatal characteristics including decreased fetal movement, will lead to younger age at diagnosis, lower burden on the healthcare system, and decreased cost (213). Improved education and outreach efforts to raise awareness of the benefits of PWS screening as part of NBS programs will be required before this can become a reality, but preliminary studies are promising (214).

### Treatment

#### Nutrition/obesity

Multiple treatment regimens are required to address the primary manifestations and comorbidities associated with PWS. One of the most immediate concerns is the regulation of nutritional intake. Infants with PWS may require nasogastric or enteric tube feeding to address poor suck and decreased caloric intake, although tube-associated risks including increased risk of reflux and scarring should be considered (181, 215). Special nipples can also be used to encourage infants to begin to feed on their own (38).

Later, hyperphagia can lead to weight gain, if not controlled. The earlier weight gain is addressed, the better the outcomes will be (216). Children with PWS benefit from highly regimented feeding schedules, including caloric restriction and dependable eating times, the latter of which can minimize food-related anxiety (31, 38, 175). Parents should also take care to eliminate food-oriented rewards and punishments. A dietitian should be consulted to ensure that proper nutritional balance is being maintained under restricted conditions (31, 39, 184, 217). Individuals should be supervised closely to prevent food-stealing, and environmental controls, such as locking refrigerators and cabinets, may be necessary. An exercise regimen should also be highly encouraged (184). Gastric bypass is not recommended for individuals with PWS, as it does not address the lack of satiety and the drive to overeat (38).

Several agents are currently in clinical trials for the treatment of hyperphagia in PWS. The anti-epileptic drug topiramate has had a significant effect on disordered eating in several small studies (218, 219) and is also recommended for the treatment of skin-picking, a common obsessive-compulsive manifestation seen in individuals with PWS (175). Several additional therapeutics with various mechanisms of action, including carbetocin, setmelanotide, diazoxide choline controlled release, unacylated ghrelin, tesofensine/metoprolol, and several glucagon-like peptide 1 receptor agonists, are currently in clinical trials for the treatment of hyperphagia and obesity (42, 220–223). Transcranial direct current stimulation trials in individuals with PWS have also shown promise in the treatment of hyperphagia (224, 225).

#### Growth/endocrine

**Figure 7:**
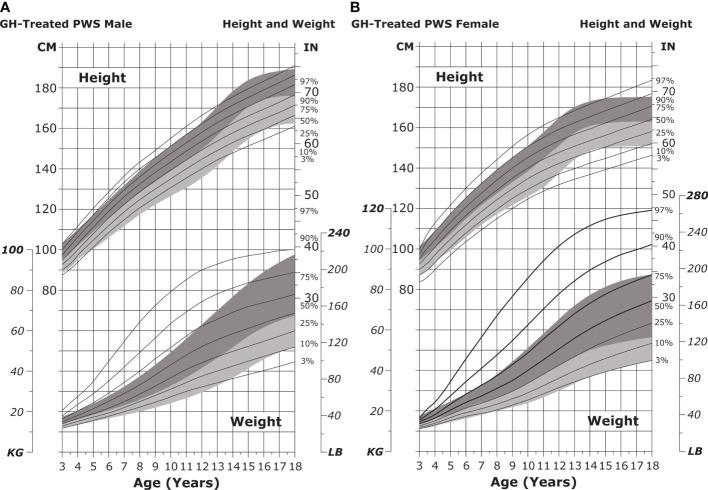
Standardized curves for height and weight of GH-treated patients with PWS. Curves for height (top) and weight (bottom) of GH-treated males **(A)** and females **(B)** with PWS. Heights and weights of GH-treated individuals with PWS are shown with solid lines and overlaid on the normative percentile ranges (shaded area) with normative 97th to 50th percentiles in dark shading and 50th to 3rd percentiles in light shading. Butler MG et al. Clin Pediatr (Phila) 2016;55(10):957-974. ^©^2016, SAGE Publications. Reprinted by Permission of SAGE Publications. GH, growth hormone; PWS, Prader-Willi syndrome.

Most individuals with PWS will exhibit GH deficiency; therefore, supplemental GH treatment is critical. GH therapy is approved in the US for individuals aged ≥2 years with PWS and documented growth failure and can normalize height outcomes by 18 years of age (**Table 3** and **Figures 7A, B**) (36, 108, 109). GH treatment can improve many additional PWS outcomes, including metabolism, body composition, mobility, behavior, bone density, and development of motor skills (36, 38, 39, 206, 226, 227). Decisions to treat with GH should be made in consultation with a pediatric endocrinologist with the understanding that earlier treatment may lead to improved outcomes (175, 216, 228). Treatment should not be started if the patient presents with severe obesity, unregulated diabetes, and/or obstructive sleep apnea (108).

GH treatment increases IGF-I concentrations, which can lead to adverse events including lymphoid tissue hypertrophy. Therefore, IGF-I should be consistently monitored in individuals treated with GH and maintained within a physiologically normal range (108). Some patients present with very high IGF-I concentrations even on low doses of GH; in these patients, treatment should be continued in the interest of growth and metabolic benefits but should be adjusted so that the ratio between IGF-I and insulin-like growth factor-binding protein 3 is maintained at baseline levels (34). In the short-term, GH therapy may increase a patient’s risk of diabetes, although this risk is likely offset by its long-term beneficial effects on body composition (228). Individuals under consideration for GH therapy should undergo a sleep study to identify obstructive sleep apnea prior to treatment initiation and periodically thereafter, as GH may worsen sleep centrally and can also cause IGF-I-induced adenotonsillar hypertrophy (38, 204). In severe cases, an otolaryngologist may recommend cessation of GH treatment until polysomnography results improve (175).

#### Puberty/genitourinary

Cryptorchidism can be treated with human chorionic gonadotropin or, if that approach fails, surgical intervention (38, 39, 175). A small study in individuals with PWS and cryptorchidism reported that 81% of testes descended to a lower position following treatment with human chorionic gonadotropin (229). Later, sex hormone replacement therapy can be helpful to stimulate development of secondary sex characteristics and to ameliorate psychosocial aspects of pubertal delay. However, sex hormone treatment is controversial, as testosterone can exacerbate behavioral problems in males and estrogen can lead to increased stroke risk and menstruation-associated hygiene concerns in females (38, 39). This risk potential needs to be evaluated in the context of delayed puberty requiring sex steroid replacement (191, 192).

#### Skeletal

Spinal deformities, including scoliosis, may result from the severe hypotonia characteristic of individuals with PWS (205). Some studies have indicated that GH treatment may contribute to worsening scoliosis in PWS; however, later studies failed to support these findings (38, 108). Physical therapy focused on core strengthening can be helpful in reducing or avoiding spinal deformities. In younger patients already presenting with a spinal curve, serial casting may be used to guide the spine into a straighter position. Bracing can be helpful in those with minor spinal curves who are too old for casting or in children who are not old enough to undergo corrective surgery (205). Surgery is typically recommended only after 12 and 14 years of age in females and males, respectively. The procedure does come with some inherent risks including the inadvertent adding-on to the spinal curve. In some cases, a combination of spinal curvature and characteristic PWS bone weakness can lead to hardware pullout or failure, which has disastrous effects (205).

#### Cognitive/behavioral

Cognitive impairments should be addressed with special educational planning and speech therapy, if needed (31, 38). Parents and patients should manage cognitive and behavioral issues, including obsessive-compulsive tendencies, anxiety, and depression, with the help of a behavioral specialist, therapist, or psychologist (175).

### Transition and multidisciplinary care

As with the other syndromes discussed in this review, early diagnosis is essential and can improve outcomes associated with PWS-related comorbidities. However, appropriate diagnosis can be difficult due to the complex testing required to identify the PWS molecular genetic class.

Treatment of individuals with PWS can be especially difficult during the transition period. While some providers may be willing and able to care for the patient with PWS from childhood through adulthood, other adult subspecialists may be unwilling to take on complex PWS cases. When a transition from pediatric to adult care is necessary, direct communication between providers (possibly at a dedicated transitional meeting) is key to ensure uninterrupted care (230). Care should overlap until all parties (providers, patients, and caregivers) are comfortable with the transition (175). Patients actively involved in the transition process are more likely to have better physical and behavioral health than those who are not involved (231).

Multidisciplinary care is essential for individuals with PWS, as they present with a plethora of co-morbidities that all require specialized care, including routine care or screening in adulthood for weight control, diabetes, hypertension, sleep apnea, peripheral edema, heart failure, and behavioral management (39, 108, 209, 231). Individuals with PWS should also receive counseling to discuss psychosexual maturation, pregnancy, and contraception (175).

Up to 20% of transition-age individuals with PWS display GH deficiency. Therefore, it is recommended that individuals transitioning to adult care undergo GH stimulation testing to inform treatment strategies during this critical time (232). Prior to medication management, pharmacogenetic testing may be considered to determine cytochrome P450 liver enzyme status, which can inform drug metabolism rates. PWS molecular genetic class may also impact the response to medication (233).

Multidisciplinary clinics can meet a real need for patients with PWS transitioning to adult health care. These clinics bring together the entire care team (geneticist, dietitian/nutritionist, endocrinologist, orthopedist, gastroenterologist, psychologist/psychiatrist, social worker, occupational therapist, and care coordinator), which can help ensure that routine medical care for an individual with PWS remains smooth and uninterrupted and can reduce morbidity for these patients. Having all providers together in one clinic can also reduce the burden on patients and their families, as multiple providers can be seen at one time and place, versus having to make different appointments for different providers in different locations (234).

The specific roles for the many members of the PWS multidisciplinary team are described below. Geneticists should perform testing to determine the molecular mechanism underlying specific cases of PWS and provide counseling on how these mechanisms may inform recurrence risk in future offspring, impact treatment for particular genetic subtypes, and affect disease surveillance (38, 39, 179, 230, 234). Endocrinologists can address GH deficiency (39), provide information on the benefits and risks of beginning GH treatment early (230, 234), and counsel patients and parents on the possibility of pubertal induction with sex hormones. Endocrinologists should be the point of contact for counseling on other PWS-associated hormonal issues such as hypothyroidism, central adrenal insufficiency, and diabetes (39, 230, 234). Long term follow up, natural history, and progression of the disorder should be undertaken by the clinical geneticist.

Nutritionists and dietitians help individuals with PWS manage nutritional intake and develop exercise plans (184). Syndrome-specific growth charts, which have been developed for patients with PWS from 0 to 18 years of age, should be used to monitor growth and development (109, 182, 235). Gastroenterologists care for patients with gastroesophageal reflux, swallowing difficulties, or gastrointestinal rupture due to overeating (39, 230, 234).

Speech pathologists can address swallowing difficulties and speech and articulation problems (31, 234). Sleep specialists are important for managing the high incidence of central and obstructive sleep apnea in individuals with PWS (175, 230, 234). Orthopedists can help to address hip dysplasia and scoliosis, with special attention given to skeletal changes that may occur during GH treatment. Ophthalmologists can help to address visual deficits or abnormalities, while dentists are important for managing PWS-associated oral problems, including caries and gingivitis (230).

Behavioral and neurodevelopmental specialists are important to address issues including social functioning, anxiety, compulsive behaviors, and developmental delays (31, 39, 175, 230, 234). Counseling for the family of an individual with PWS is also key, as the plethora of comorbidities associated with this syndrome can take a mental and emotional toll on everyone involved.

Finally, the importance of the care coordinator should not be understated. Care coordinators may play a variety of roles and may be responsible for tasks as diverse as helping patients attend their appointments, improving communication between health care providers, educating patients and families, and assisting with insurance issues (234).

## Discussion/conclusion

The three syndromes discussed here are genetic disorders that affect height outcomes and, typically, GH sensitivity (Noonan syndrome, Turner syndrome) or production (some cases of Noonan syndrome, most cases of Prader-Willi syndrome) (**Table 1**). Although the syndromes may differ in their phenotypic presentations (**Table 1**), nearly all patients will present with short stature and may benefit from GH supplementation at some point in their lives.

GH treatment may be associated with physical and emotional challenges. Painful daily injections and fear of side effects may decrease treatment adherence in a small percentage of patients (236–238). The doses themselves can also constitute a burden, as maintaining proper storage, handling, and reconstitution of the product may be complicated, particularly during travel (236–238). Some patients report emotional consequences of daily GH injections, including fear of injections and feeling different from others because of their treatment (239). However, the authors of this review note that these fears are by no means universal and are not typically observed in their own clinical practice.

GH treatment in short children demonstrably improves patients’ physical, social, and emotional quality of life (240). Further, patient concerns may, in some cases, be balanced by the desire to achieve the best possible growth outcomes (239).

Current GH treatments are given as daily subcutaneous injections. However, in recent years, long-acting GH (LAGH) therapies have been developed that may reduce treatment burden and increase adherence. LAGH therapies may be patient-preferred (238), as they require less frequent injections; however, these molecules may display suboptimal tissue distribution as a result of the modifications required to extend their half-life (e.g., PEGylation) (241). Currently, two LAGH formulations, somapacitan and lonapegsomatropin, are FDA-approved for use in adult and pediatric GH deficiency, respectively (242). Studies are ongoing to evaluate the benefit of other LAGH formulations in additional indications such as TS (243–245).

NS, TS, and PWS are all accompanied by comorbidities that require a lifetime of monitoring and treatment as described. Therefore, it is important for individuals with any of these syndromes to receive care from a multidisciplinary team of specialists throughout their life span. However, several factors, including diagnostic delays and a lack of communication among health care specialists, can complicate the comprehensive care needed to support patients with these disorders.

It can be difficult for patients to access high-quality care from multiple specialists, especially if they live in geographically isolated areas or have financial constraints. In today’s increasingly connected world, telemedicine can be a great option to ensure that patients consult with all their care providers and to increase communication between these providers (234).

Here, we present the clinical features and genetics associated with each disorder, diagnostic techniques, and treatment methods. We also discuss the importance of multidisciplinary care and proper, supported transition from pediatric to adult health care systems with the hope that this discussion will reinforce best practices when diagnosing and treating patients with NS, TS, and PWS.

## Author contributions

All authors contributed to the literature search, drafting, revisions, and editing of the manuscript. All authors approved the final version of the manuscript. 

## Funding

Novo Nordisk Inc. (Plainsboro, NJ) financial support was limited to medical writing support, as indicated in the Acknowledgments section.

## Acknowledgments

Medical writing support was provided by Amy Ross, PhD, of PRECISIONscientia (Yardley, PA) in compliance with international Good Publication Practice guidelines and was supported financially by Novo Nordisk. Nicky Kelepouris, MD, of Novo Nordisk, provided a medical accuracy review of the manuscript.

## Conflict of interest

AR is a speaker and consultant for Novo Nordisk and a consultant for Ascendis Pharma. JR is a consultant and has research support from Novo Nordisk and is a consultant with OPKO. MA receives research support from Ascendis, Levo, Lumos, Novo Nordisk, Rhythm, and Soleno. MA has been on advisory boards for Pfizer and Rhythm. PB has been a consultant for Novo Nordisk, Novartis/Sandoz, Tolmar, Ascendis Pharma, BioMarin, Cavalry Bioventures, and Ipsen, and currently receives research support from Novo Nordisk and Ipsen. NM has received institutional research grants from Novo Nordisk and OPKO and has received consulting fees from Agios.

The remaining authors declare that the research was conducted in the absence of any commercial or financial relationships that could be construed as a potential conflict of interest.

## Publisher’s note

All claims expressed in this article are solely those of the authors and do not necessarily represent those of their affiliated organizations, or those of the publisher, the editors and the reviewers. Any product that may be evaluated in this article, or claim that may be made by its manufacturer, is not guaranteed or endorsed by the publisher.

## Footnotes

1
https://rasopathiesnet.org/wp-content/uploads/2014/01/265_Noonan_Guidelines.pdf.

2
https://rarediseases.org/rare-diseases/noonan-syndrome/.

3
https://bestpractice.bmj.com/topics/en-us/1193.

4
https://www.acponline.org/system/files/documents/clinical_information/high_value_care/clinician_resources/pediatric%20_adult_care_transitions/endo_turner/endo_ts_transition_tools.pdf.

## Abbreviations

ADHD, attention deficit hyperactivity disorder; ASD, autism spectrum disorder; CFC, cardiofaciocutaneous; CHD, congenital heart disease; FDA, Food and Drug Administration; GH, growth hormone; HCM, hypertrophic cardiomyopathy; IGF-I, insulin-like growth factor I; LAGH, long-acting growth hormone; MAPK, mitogen-activated protein kinase; MRA, magnetic resonance angiography; MRI, magnetic resonance imaging; NBS, newborn screening; NF1, neurofibromatosis type 1; NORD, National Organization for Rare Disorders; NS, Noonan syndrome; NS/MPD, Noonan syndrome/myeloproliferative disorder; PS, pulmonary stenosis; PWS, Prader-Willi syndrome; SDS, standard deviation score; TRAQ, Transition Readiness Assessment Questionnaire; TS, Turner syndrome; UPD, uniparental disomy.
